# Chondroitinase and Growth Factors Enhance Activation and Oligodendrocyte Differentiation of Endogenous Neural Precursor Cells after Spinal Cord Injury

**DOI:** 10.1371/journal.pone.0037589

**Published:** 2012-05-22

**Authors:** Soheila Karimi-Abdolrezaee, Desiree Schut, Jian Wang, Michael G. Fehlings

**Affiliations:** 1 Regenerative Medicine Program and Department of Physiology, University of Manitoba, Winnipeg, Canada; 2 Toronto Western Research Institute, Toronto, Canada; 3 Department of Surgery and Neuroscience Program, University of Toronto, Toronto, Canada; University of Nebraska Medical Center, United States of America

## Abstract

The adult spinal cord harbours a population of multipotent neural precursor cells (NPCs) with the ability to replace oligodendrocytes. However, despite this capacity, proliferation and endogenous remyelination is severely limited after spinal cord injury (SCI). In the post-traumatic microenvironment following SCI, endogenous spinal NPCs mainly differentiate into astrocytes which could contribute to astrogliosis that exacerbate the outcomes of SCI. These findings emphasize a key role for the post-SCI niche in modulating the behaviour of spinal NPCs after SCI. We recently reported that chondroitin sulphate proteoglycans (CSPGs) in the glial scar restrict the outcomes of NPC transplantation in SCI by reducing the survival, migration and integration of engrafted NPCs within the injured spinal cord. These inhibitory effects were attenuated by administration of chondroitinase (ChABC) prior to NPC transplantation. Here, in a rat model of compressive SCI, we show that perturbing CSPGs by ChABC in combination with sustained infusion of growth factors (EGF, bFGF and PDGF-AA) optimize the activation and oligodendroglial differentiation of spinal NPCs after injury. Four days following SCI, we intrathecally delivered ChABC and/or GFs for seven days. We performed BrdU incorporation to label proliferating cells during the treatment period after SCI. This strategy increased the proliferation of spinal NPCs, reduced the generation of new astrocytes and promoted their differentiation along an oligodendroglial lineage, a prerequisite for remyelination. Furthermore, ChABC and GF treatments enhanced the response of non-neural cells by increasing the generation of new vascular endothelial cells and decreasing the number of proliferating macrophages/microglia after SCI. In conclusions, our data strongly suggest that optimization of the behaviour of endogenous spinal NPCs after SCI is critical not only to promote endogenous oligodendrocyte replacement, but also to reverse the otherwise detrimental effects of their activation into astrocytes which could negatively influence the repair process after SCI.

## Introduction

Spinal cord injury (SCI) results in limited spontaneous axonal remyelination [1], [2], [3] despite the existence of multipotent endogenous spinal neural precursor cells (**es**NPCs) with the potential to replace lost oligodendrocytes [4], [5], [6], [7]. Although increasing evidence demonstrates that SCI triggers the proliferation of esNPCs in the ependymal layer 6,8,9,10,11, the outcome of this activity does not result in a sizable and meaningful oligodendroglial differentiation and maturation following SCI. Activated esNPCs from the ependymal region predominantly differentiate into astrocytes after SCI and generation of oligodendrocytes essential for remyelination is very limited [6], [7], [10], [12]. The newly generated astrocytes migrate to the site of injury, become reactive and contribute to astrogliosis after SCI [10], [13]. This evidence suggests that the cell replacement potential of esNPCs is negatively influenced by their post SCI-environment. Optimization of the behaviour of esNPCs after SCI is important not only to promote endogenous oligodendrocyte replacement, but also to reverse the otherwise non-constructive effects of their activation in generating glial scar and inhibiting repair and regeneration after SCI.

Following SCI, neuroinflammation and reactive astrogliosis profoundly modify the extracellular matrix (ECM) and foster the generation of an inhibitory environment to neural repair and regeneration [14]. Chondroitin sulfate proteoglycans (CSPGs) are up-regulated in the ECM around the SCI lesion and exert significant inhibitory effects on spinal cord regeneration and plasticity [15], [16], [17], [18], [19]. In a rat model of chronic compressive SCI, we have recently shown that the up-regulation of CSPGs potently limit the survival, integration and migration of transplanted adult NPCs [18]. When we targeted CSPGs with Chondroitinase ABC (ChABC) at chronic stages of SCI and prior to NPC transplantation, it significantly enhanced the survival, mobilization and integration of engrafted NPCs within the chronically injured spinal cord [18]. The effects of ChABC was furthered enhanced by combining ChABC with the transient administration of a cocktail of growth factors that included epidermal growth factor (EGF), basic fibroblast growth factor (bFGF) and platelet derived growth factor-AA (PDGF-AA) [18]. The combined effects of ChABC and growth factors enhanced the long-term survival of transplanted NPCs and directed their differentiation along an oligodendroglial lineage associated with remyelination and functional recovery in a chronic model of SCI [18].

In the present study, we addressed whether optimizing the injury microenvironment with combination of ChABC and the growth factors (GFs) would promote the regenerative response of esNPCs early after SCI. Here, we show for the first time that perturbing CSPGs and increasing the bioavailability of key growth factors required for the expansion of NPCs significantly improved the proliferation of esNPCs and decreased their differentiation into astrocytes which was also accompanied by reduced inflammation and reactive astrogliosis and increased generation of new endothelial cells in the injured spinal cord. Importantly, combined effects of ChABC and GFs enhanced oligodendroglial differentiation of esNPCs after SCI. These results suggest that optimizing the inhibitory microenvironment for spinal cord precursor cells is a promising regenerative approach for SCI, which offers a potential alternative or complementary approach to cell transplantation.

## Methods

### Animals and Animal Care

All experimental protocols were approved by the Animal Committee of the University of Manitoba and the University Health Network in accordance with the policies established in the guide to the care and use of experimental animals prepared by the Canadian Council of Animal Care. A total of 55 adult female Wistar rats (250 g; Charles River Laboratories, Quebec, Canada) were used in this study. Animals were housed in standard plastic cages at 22°C before spinal cord injury (SCI) and 26°C after SCI in a 12∶12 h light/dark photoperiod. Pelleted food and drinking water were available *ad libitum*. Hardwood sawdust bedding was used before SCI and then was replaced by soft paper bedding after SCI to prevent skin lesion. After surgeries, animals were given buprenorphine (Temgesic, 0.05 mg/kg) three times every 8 hrs to manage pain and discomfort. SCI rats were examined daily and the bladder was expressed three times daily until the recovery of spontaneous bladder function.

### Rat Compressive Thoracic Spinal Cord Injury Model

We used an aneurysm clip compression model of SCI that has been extensively characterized and used by our group [1], [18], [20], [21], [22], [23]. All surgeries were performed under aseptic conditions and using general gas anesthesia consisting of a mixture of O_2_/isoflurane (1–4%) given through a mask integrated in a surgical stereotaxic frame. The surgical area was shaved and disinfected with 70% ethanol and betadine. A midline incision was made at the thoracic area (T4–T9) and skin and superficial muscles were retracted. The rats underwent a T6–T8 laminectomy and then, received a 23 g clip (Walsh Inc., Oakville, Ontario, Canada) compression injury for 1 min at the level of T7 of the spinal cord. The surgical wounds were sutured and the animals were given postoperative analgesia (Temgesic, 50 µg/kg) and saline (0.9%; 5 ml) to prevent pain and dehydration. Animals received Clavamox (Amoxicillin plus Clavulanic Acid, Pfizer Animal Health) for 5 days. The model characterization, histological assessment of injury and behavioral assessment have been characterized extensively by our group [1], [18], [20], [21], [24].This model of SCI in the rat accurately mimics the key features of human SCI. It creates a model of moderately severe SCI, which results in a central cavitation and loss of 80% of axons in the spinal cord white matter (Fehlings and Tator, 1995), demyelination of the surviving axons in the residual white matter as well as unmyelinated axons in the injured spinal cord and behavioral evidence of a spastic paraparesis (Nashmi et al., 1997; Nashmi and Fehlings, 2001). Collectively, the outcome of this model is of relevance to the majority of patients with severe, incomplete SCI.

### Experimental Groups and Treatments

Before SCI, rats were randomly allocated into four experimental groups to receive either vehicle or ChABC and/or growth factor treatments. The experimental groups included: 1) Injured-vehicle (receiving vehicle containing saline plus 0.1% rat serum albumin), 2) Injured-ChABC (receiving chondroitinase ABC, 5 U/ml in vehicle, Seikagaku Corporation, Tokyo, Japan), 3) Injured-Growth Factors (a GFs cocktail containing including PDGF-AA (Sigma, 1 µg/100 µl), bFGF (Sigma, 3 µg/100 µl) and EGF (Sigma, 3 µg/100 µl) in vehicle), and 4) Injured-ChABC+GFs (receiving the combination of ChABC and GFs in vehicle). A summary of our experimental approaches and groups is depicted in Table S1.

#### Administration of treatments

At four days post SCI, the injured rats were anesthetized using isoflurane inhalation (1–2%) and a 1∶1 mixture of O_2_/N_2_O and then the injured spinal cord was carefully re-exposed using microsurgical techniques. Treatments were administered intrathecally using a fine catheter (Alzet, Rat IT, 0007741, 0.36 mm OD; 0.18 mm ID) connected to an osmotic mini-pump (Alzet pump model No.1007D, 0.5 µl/hr) for 7 days as we reported previously [18], [21]. The catheter was securely inserted in the subarachnoid space around the injured area.

#### BrdU incorporation regimen

To examine the proliferation kinetics and fate of the mitotically active cells in the injured spinal cord after treatments, all rats received 5-Bromo-2-deoxyuridine (BrdU, IP, 50 mg/kg) injections starting on the treatment day. The rats received two daily injections of BrdU and a last one at 2 hours before euthanasia for a of total 15 injections.

### Euthanasia/tissue Processing

Seven days after treatments (12 days post-SCI), rats in all treatment groups were deeply anesthetized with isoflurane inhalation and then perfused transcardially with cold PBS (0.1 M) followed by 4% paraformaldehyde (PFA) in 0.1 M PBS, pH 7.4. A 3 cm length of the spinal cord centered at the injury center was dissected and postfixed in the perfusing solution plus 10% sucrose overnight at 4°C, and then cryoprotected in 20% sucrose in PBS for 48 h at 4°C. Then, the spinal cord centered at the injury site was dissected and embedded in mounting media (HistoPrep, Fisher Scientific) on dry ice. Cryostat sections (25 µm) were cut and stored at -70°C.

### Histological Assessment of SCI Lesion

To examine the spinal cord lesion and determine the epicenter of SCI, we used Luxol Fast Blue (LFB) and hematoxylin-eosin (HE). Tissue sections displaying the largest proportion of tissue disintegration compared with total cross-sectional area were taken to represent the epicentre of the injury.

### Immunohistochemical Procedures and Image Analysis

For all immunohistochemical staining, the blocking solution contained 5% non-fat milk, 1% BSA, and 0.3% Triton X-100 in 0.1 M PBS unless otherwise has been mentioned.

#### Immunodetection of GFAP, CSPGs, and C4S-DS

The frozen slides were air dried at room temperature for 20 min and then were washed with PBS for 10 min. The sections were blocked and then incubated with primary antibodies. The following primary antibodies were used overnight at 4°C: rabbit anti-GFAP (1∶1000, Millipore Bioscience Research Reagents) for astrocytes, mouse anti-CSPGs (CS56, Sigma, 1∶200), mouse anti-chondroitin-4-sulfate (C4S, 1∶200, Millipore Bioscience Research Reagents) for visualization of CSPG GAG digestion. The slides were washed in PBS three times and then incubated with fluorescent Alexa 568 or 488 or 647 goat-anti mouse secondary antibodies (Invitrogen, 1∶400) for 1 h. The slides were coverslipped with Mowiol mounting medium containing DAPI to counterstain the nuclei. The images were taken using a Zeiss 510 laser confocal microscope or Leica epifluorescence microscope.

For immunoblotting, spinal cords were dissected, placed in ice-cold artificial CSF (124 mM NaCl, 3 mM KCl, 1 mM NaHPO4, 26 mM NaHCO3, 1.5 mM MgSO4, 1.5 mM CaCl2, and 10 mM glucose), and cleaned from meninges and nerve roots. One centimeter of the cord centered at the injury site was dissected and homogenized (in a buffer composed of 50 mM Tris-HCl, pH 7.4, 1****mM EDTA, 0.1% SDS, 100 µM leupeptin, 1 µM pepstatin, 10 µg/ml aprotinin, and 100 µM phenylmethylsulfonyl fluoride). For slot blotting, 3 µg of protein/well was transferred to polyvinylidene difluoride membranes (Millipore) using Bio-Dot slot blot apparatus (Bio-Rad) according to the manufacturer’s instructions. The membranes were then incubated with mouse anti-CSPGs (CS-56, 1∶300, Sigma), mouse anti-GFAP (MAB360, Chemicon, 1∶5000), mouse anti-C4S (Chemicon, 1∶200) and rabbit anti-Iba1 (016-20001, Wako, 1∶1000). Following the incubation with HRP-conjugated goat anti-mouse antibody (1∶5000), membranes were incubated in ECL plus immunoblotting detection reagents (GE Healthcare) according to the manufacturer’s specifications. Immunoreactive bands were quantified using Gel-Pro Analyzer software (Media Cybernetics). To control for equal protein loading, membranes were also probed for actin and CSPG values were normalized to actin (1∶200, Millipore Bioscience Research Reagents). Loading BSA (bovine serum albumin) was used as a negative protein control.

#### Multiple immunofluorescence labeling

To assess the phenotype of BrdU+ cells triple labelling immunofluorescence was employed. The frozen slides of injured rats were blocked and sequentially incubated with antibodies to mark two neural markers as well as BrdU. Since some immunolabels are not specific to a single cell type, we carefully selected triple immunolabeling combinations to account for the possible overlap. A detailed list of triple labelling immunostaining paradigms and the type of primary antibodies used in our studies is provided in Tables 1 and 2. After incubation with each primary antibody, the slides were washed in PBS three times and incubated with appropriate secondary fluorescent Alexa 568, 488 or 647 (1∶400; Invitrogen) for 1 h. The slides were washed and coverslipped with Mowiol mounting medium containing DAPI. For BrdU detection, the tissue was pre-treated with 2 N HCl for 30 minutes at 37°C and then washed with sodium borate buffer for 10 minutes followed by two PBS wash before incubating with BrdU antibody.

**Table 1:** Antibodies.

Antibody	Specificity	Source	Dilution factor
BrdU	Proliferating cells that have incorporated BrdU in S phase	Serotec (Rat, OBT0030G)	1∶400
Nestin	Neural stem/progenitor cells	Chemicon (Mouse, MAB353)	1∶200
GFAP	astrocytes	Chemicon (Mouse, MAB360)	1∶800
GFAP	astrocytes	Chemicon (Rabbit, AB5804)	1∶1000
CS56	CSPGs	Sigma (Mouse, C8035)	1∶200
C4S	Chondroitin 4 sulphate	Chemicon (Mouse, MAB2030)	1∶200
OX42	Macrophages/microglia	Serotec (Mouse, MCA275G)	1∶50
Iba-1	Macrophages/microglia	Wako (Rabbit, 019-19741) For immunostaining	1∶500
Iba-1	Macrophages/microglia	Wako (Rabbit, 016-20001) For immunoblotting	1∶1000
Olig2	Oligodendrocyte progenies	Chemicon (Rabbit, AB9610)	1∶1000
NG2	Oligodendrocyte precursor cells	Chemicon (Rabbit, AB5320)	1∶200
NG2	Oligodendrocyte precursor cells	Chemicon (Mouse, MAB5384)	1∶200
APC (CC1)	Mature oligodendrocytes	Calbiochem (Mouse, OP80)	1∶50
Reca-1	Endothelial cells	Serotec (Mouse, MCA970R)	1∶25
Actin	cytoskeleton filament (housekeeping protein)	Chemicon (Mouse, MAB1501R)	1∶300

**Table 2:** Summary of triple immunolabeling cell quantifications.

Multiple labelling combinations	Cell type and criteria for cell counting
Nestin/GFAP/BrdU	Nestin+/GFAP+/BrdU+: considered as astrocyte
	Nestin−/GFAP+/BrdU+: considered as astrocytes
	Nestin+/GFAP−/BrdU+: considered as NPCs
Olig2/GFAP/BrdU	Olig2+/GFAP+/BrdU+: considered as astrocytes
	Olig2+/GFAP−/BrdU+: considered as oligodendrocyte progenies
Olig2/NG2/BrdU	Olig2+/NG2+/BrdU+: considered as oligodendrocyte precursors
Olig2/APC/BrdU	Olig2+/APC+/BrdU+: considered as mature oligodendrocyte
Reca-1/NG2/BrdU	Reca-1+/NG2+/BrdU+: considered as endothelial cells
Iba-1/NG2/BrdU	Iba-1+/NG2+/BrdU+: considered as macrophages/microglia

#### Confocal microscopy and image analysis

We used confocal microscopy to acquire images of the multi-fluorescent labelled sections of the spinal cord. For quantification of cell proliferation and differentiation, we examined one cross section of the spinal cord between 2–3 mm rostral and caudal to the injury center where all injured rats had an intact central canal and ependymal layer (n = 5–6 rats/group). We immunostained the spinal cord sections for six combinations of cell markers and BrdU as shown in Table 1. Using images taken by confocal microscopy at 25× primary magnification, we first counted the number of BrdU-positive cells in 6 specified (368 µm×368 µm) per spinal cord cross section. The selected fields included the ependymal/subependymal region, dorsal and lateral columns and ventral gray and white matter. Then, we calculated the percentage of colabeled BrdU/cell markers for each triple labelling combination (summarized in Table 2) in each spinal cord section. In all neuroanatomical procedures, quantification was executed in a stereologically unbiased manner by examiners blinded to the treatment groups based on the previously described methods by our group [18], [21].

### Statistics and Randomization

Unbiased assessments were undertaken using appropriate randomization and blinding in all experimental protocols. Statistical analyses of intensity measurements and cell counts were tested by one-way ANOVA comparing groups followed by *post hoc* pairwise multiple-comparison testing by the Holm–Sidak method or by Student’s *t* tests where two groups were compared. Data are reported as means ±SEMs, and *p*<0.05 was considered significant.

## Results

### Integrity of Ependymal Layer After Compressive SCI in Rat

**Figure 1:**
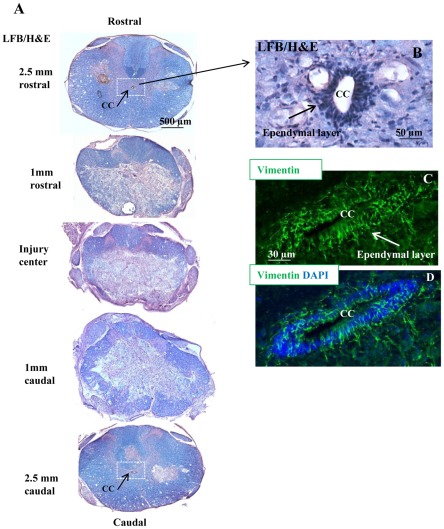
Representative images of compressive SCI in rats at 12 days post-injury. (A) Images of thoracic spinal cord cross sections at various rostral and caudal distances to the injury center were counter-stained with Luxol Fast Blue (LFB) and hematoxylin/eosin (H&E). We found the presence of an intact central canal (CC) at around 2.5 mm away from the injury center in all rats. (B) Magnified area in the box in (A) show the ependymal layer that lines the central canal. (C-D) Confocal images of the central canal immunostained for vimentin.

In this study, we used an aneurysm clip compression model of SCI in the rat that closely mimics the hallmarks of human SCI neuropathology [1], [18], [21], [22], [23]. We utilized a 23 g injury force that results in an incomplete and moderately severe SCI. We have extensively characterized and used this model in a number of pharmacological and cellular therapeutic approaches [1], [18], [20], [21], [25]. The rat SCI model results in inflammation, astrogliosis, neuronal and oligodendroglial cell death, axonal degeneration and demyelination that collectively lead to significant spinal cord tissue loss and consequently the formation of a central cavity in the chronic stage of injury (i.e. around four weeks after SCI). Adult esNPCs mainly reside in the ependymal region lining the central canal of the spinal cord [6], [7], [11] (Fig. 1B–D). Because of their central localization in the spinal cord, they are directly subjected to the cell loss after SCI. To characterize the integrity of ependymal region in the rat compressive SCI, we performed Hematoxylin/Eosin staining at various distances to the injury center. At 12 days after SCI, the time point that we collected the spinal cord tissues after treatments, we found an intact ependymal region at approximately 2.5 mm rostral and caudal to the injury center in all injured animals (Fig. 1). Accordingly, we undertook our tissue phenotypic assessments in a distance between 2.5 to 3 mm from the injury center to ensure the presence of esNPCs in the ependymal region in all animals.

### CSPG Degradation after ChABC Treatment

**Figure 2:**
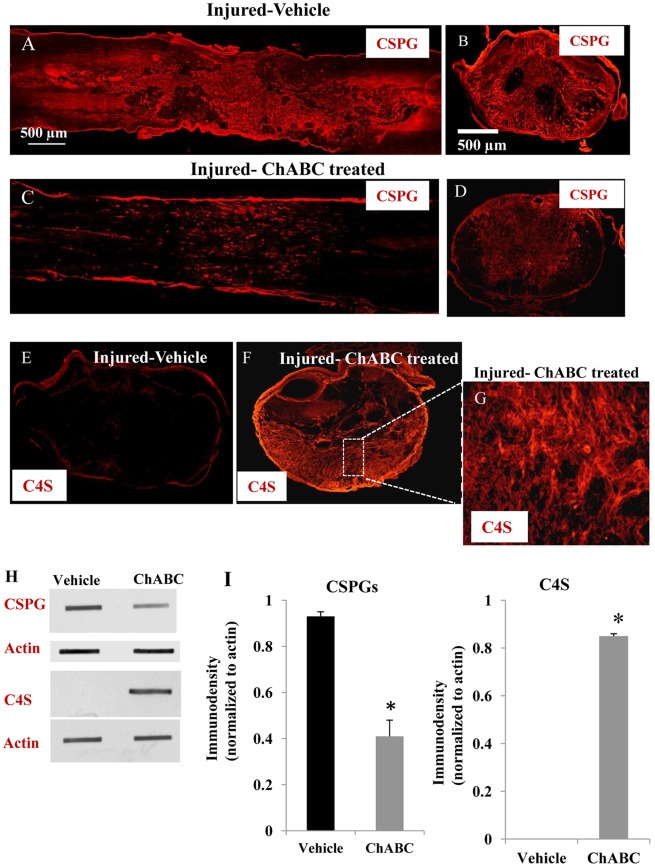
ChABC efficiently degrades CSPG in the injured spinal cord. (A, B) Confocal images of horizontal and cross sections of vehicle treated spinal cords 12 days after SCI shows the intense expression of CSPGs (red) at the lesion site. (C, D) Treatment with ChABC at 4 days post-injury for one week substantially reduced CSPGs deposits in the injured spinal cord. (E-G) Successful degradation of CSPGs was further confirmed by pronounced appearance of chondroitin- 4-sulfate (C4S) in ChABC treated rats, which marks the tetrasaccharride linker region, or stub, that results from ChABC enzymatic action. Inset image (E) represents the enlarged box in F that shows a magnified area of C4S immunoreactivity in ChABC treated-injured rats. (H, I) Degradation of CSPGs by ChABC was confirmed by slot blotting. Quantification of CSPGs revealed a significant reduction in CSPG level after ChABC treatment compared to the vehicle treated injured rats. This was supported by dramatic appearance of C4S immunoreactivity in slot blotting in ChABC treated injured rats with no apparent immunoreactivity in vehicle group. (*p<0.05, n = 3/group).

We examined the effects of SCI-induced up-regulation of CSPGs on the proliferation and differentiation pattern of spinal cord precursors by using ChABC as we described previously [18]. We administered ChABC intrathecally at four days post SCI when dense deposits of CSPGs accumulate in the ECM around the spinal cord lesion [26]. We also chose to perform a delayed subacute ChABC treatment in our studies in the light of evidence that shows inhibition of CSPG synthesis immediately after injury seems to exacerbate the outcomes of SCI by changing the inflammatory response [27]. After eight days of sustained ChABC treatment, using slot blot and immunohistochemistry analyses against anti-chondroitin sulfate CS56 antibody that recognizes the intact GAG side chains of CSPGs, we found significant reduction in expression of CSPGs in ChABC-treated injured rats (*n* = 6) compared to the vehicle-treated counterparts (*n* = 6) (Fig. 2). Although ChABC did not entirely remove CSPGs, we found a 2.9-fold reduction in the deposits of CSPGs around the SCI lesion compared to the vehicle treated rats as evident by slot blot analysis (Fig. 2H–I). We further confirmed enzymatic degradation of CSPGs by immunohistochemistry against C4S, which marks the tetrasaccharide linker region, or stub, that results from ChABC enzymatic action. With no apparent endogenous C4S immunoreactivity in non-ChABC-treated injured animals (Fig. 2E–G), rats treated with ChABC exhibited a pronounced expression of C4S in the spinal cord tissue surrounding the lesion site (Fig. 1H–I).

### The Effects of ChABC and Growth Factor Treatments on Cellular Proliferation after SCI

**Figure 3:**
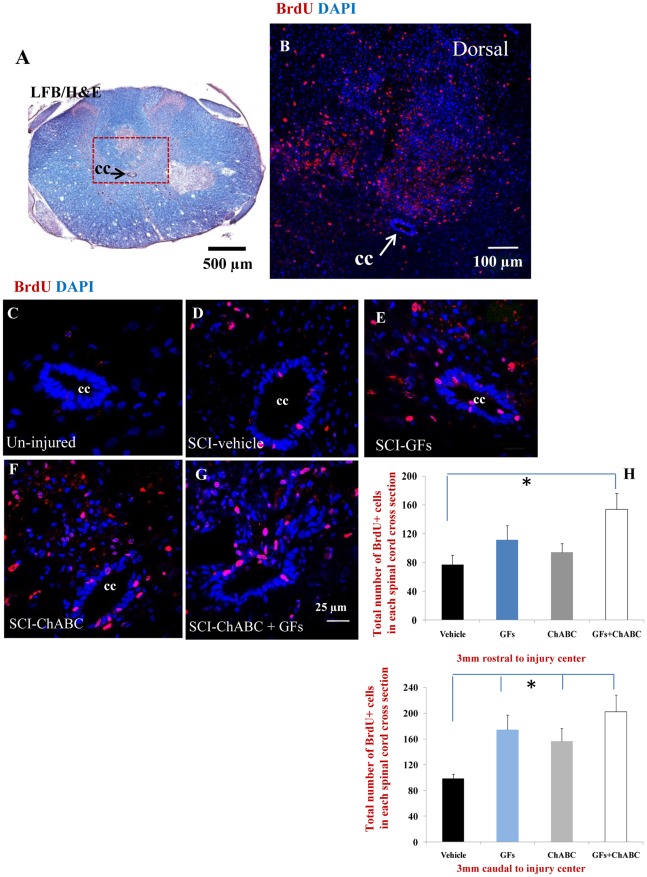
Increased number of proliferating cells after ChABC and GF treatments. (**A–B**) Representative cross sections of a subacutely injured spinal cord at 12 days after SCI. At approximately 2.5 mm rostral point to the injury center, there was a significant number of BrdU labelled cells within the injured spinal cord particularly in the dorsal column. (B) Higher magnification of the boxed area in (A) shows the presence of BrdU labelled cells (red). (C–G) BrdU immunohistochemistry showed rare proliferative activity inside the spinal cord under normal condition (C). Our results showed an increase in the number of BrdU-labeled cells (evidence of proliferation) after SCI under baseline condition (D). Interestingly, GF and/or ChABC treatments were able to enhance cell proliferation in the areas distant to the injury center (E–G). The effect was significant for ChABC+GF treated group in the rostral point and for all treatment groups in the zone caudal to the lesion epicentre compared to the vehicle group (H). *p<0.05, n = 6/group.

We studied cell proliferation after SCI by using the BrdU incorporation method. We administered BrdU daily during the treatment period to label all dividing cell populations within the injured spinal cord. Using confocal immunohistochemistry, we found a significant number of BrdU labelled cells in the injured spinal cord tissue (Fig. 3B) compared to the un-injured spinal cord which only contained a very few BrdU^+^ cells mostly around the ependymal region (Fig. 3C). In the injured animals, BrdU^+^ cells were scattered in the entire cross sectional area of the spinal cord tissue with a more pronounced density in the dorsal column (Fig. 3A–B). We quantified the number of BrdU^+^ cells in the cross sections of the spinal cord under different treatments at 2.5–3 mm point to the injury center both rostrally and caudally where all animals had an intact central canal and ependymal region (Fig. 1A). Our treatment groups consisted of: 1) vehicle-treated (representing SCI baseline), 2) ChABC-treated, 3) GF-treated and 4) ChABC+GF treated. Using imaged taken from a confocal microscope, we counted BrdU^+^ cells in six defined areas of the spinal cord that included ependymal/subependymal region and central gray matter, ventral gray and white matter, as well as lateral and dorsal white matter. Our cell quantification analysis revealed an overall increase in the number of BrdU^+^ cells in all treatment groups (ChABC, GFs and ChABC+GFs) compared to the vehicle-treated group in both rostral and caudal points to the injury center (Fig. 3H). The increased number of BrdU^+^ cells was significant for all treatment groups caudal to the lesion epicentre. In the rostral regions, although all groups showed a trend for increased number of proliferating cells in the spinal cord, only the value for ChABC+GFs group reached the level of significance level (p<0.05, Fig. 3H).

### ChABC and Growth Factor Treatments Increase the Proliferation of Nestin Positive esNPCs After SCI

**Figure 4:**
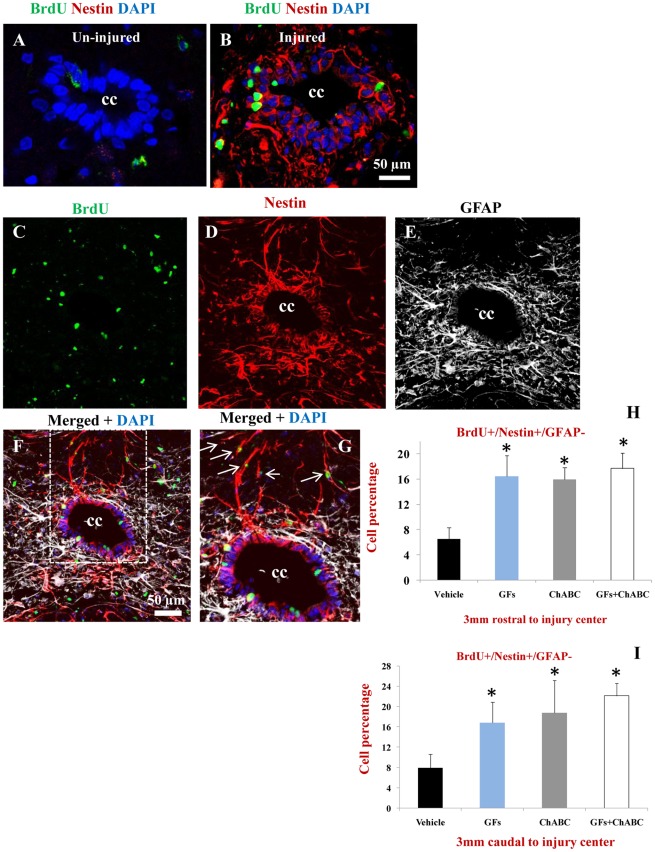
ChABC and GFs enhance the proliferation of NPCs after SCI. (A–B) Confocal images showing ependymal region in the central canal (CC) of the spinal cord. After SCI (B), there was a significant increase in the expression of nestin (red) in the ependymal/subependymal cells compared to the un-injured spinal cord with no apparent nestin immunostaining (A). SCI also induces the proliferation of ependymal cells evident by BrdU labelling (green). (C–G) Representative confocal images of the ependymal region of a rat treated with ChABC+GFs. Triple labelling for Nestin/GFAP/BrdU was used to identify proliferating/activating NPCs marked as BrdU+/nestin+/GFAP-. This immunostaining combination was used to distinguish nestin+ NPCs from nestin positive reactive astrocytes after SCI (Table 2). Normal astrocytes (GFAP+) and reactive astrocytes were mostly confined in the subependymal layer whereas Nestin+/GFAP- cells resided in the ependymal layer (C–G). We found BrdU+/nestin+/GFAP- that were migrating away from the ependymal layer to reside in parenchyma (arrows in G) Our quantitative confocal immunohistochemistry revealed a significant number of BrdU+/nestin+/GFAP- (in all treatment groups compared to the vehicle-injured group particularly around the central canal at both 3 mm rostral and caudal points to the injury center (H-I, *p<0.05, n = 6/group). **Note:** Our immunolabeling showed the absence of GFAP expression in ependymal cell. Although GFAP positive astrocytes were closely surrounding the ependymal layer and sending their process into the region, no GFAP expressing cell was observed inside the ependymal layer.

After SCI, we found a dramatic expression of nestin in cells residing in the ependymal layer and subependymal region, whereas no apparent nestin expression was present in the un-injured spinal cord (Fig. 4A–B). We next examined whether ChABC and/or GFs treatments enhanced the proliferation of nestin positive precursor cells in the spinal cord. Because reactive astrocytes also express nestin after SCI, we employed triple labelling staining combination against nestin/GFAP/BrdU to distinguish proliferating esNPCs from reactive astrocytes. We considered activated esNPCs as BrdU+/Nestin+/GFAP- and proliferating reactive astrocytes as BrdU+/Nestin+/GFAP+ (Table 2). Using this criterion, we found a significant increase in the number of newly proliferating esNPCs (BrdU+/Nestin+/GFAP-) cells in all treatment groups in comparison to the vehicle-treated rats both in rostral and caudal points to the injury center (Fig. 4I, 4J).

### Combination of ChABC and Growth Factors Promote Oligodendrocyte Differentiation After SCI

**Figure 5:**
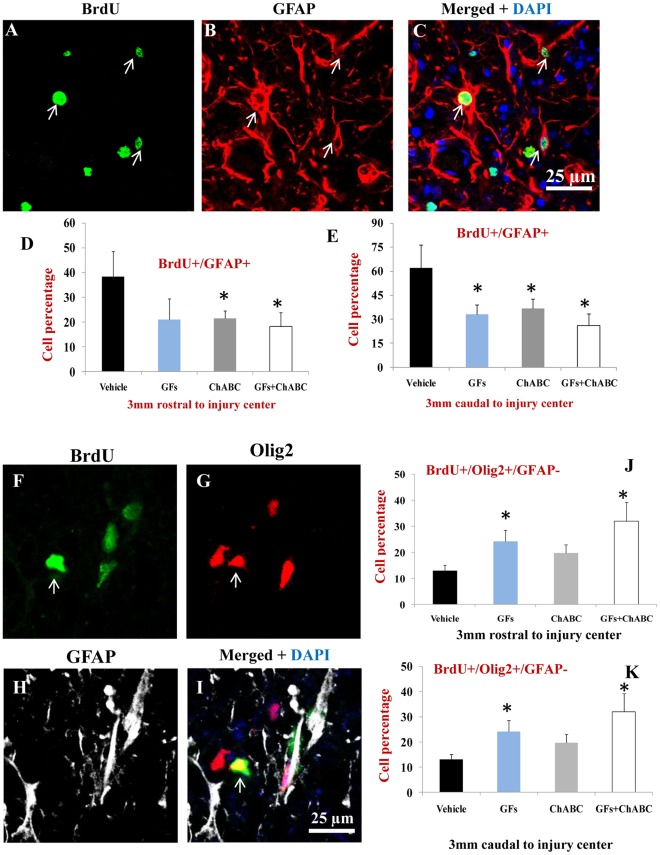
ChABC and GFs reduce astrocyte differentiation and promote the formation of new oligodendrocytes after SCI. (A–C) Representative confocal images showing BrdU labelled astrocytes that were newly generated after treatments in the injured spinal cord. (D–E) Our cell quantification for BrdU+/GFAP+ profiles (arrows in C) showed a significant decrease in the number of new astrocytes after treatment with ChABC and ChABC+GFs compared to the vehicle group in the 3 mm rostral point to the injury center. We also found a significant reduction in astrocyte proliferation in all treatment groups at 3 mm caudal distance to the injury center relative to the vehicle group (D, E, *p<0.05, n = 6/group). (F–H) Representative confocal images show triple labelling for BrdU/Olig2/GFAP (arrows). To quantify BrdU labelled cells with oligodendrocyte phenotype, we only quantified Olig2+/GFAP− to exclude Olig2 expressing reactive astrocytes. Interestingly, there was a significant increase in the number of newly generated oligodendrocytes (arrows) in GFs and ChABC+GF in comparison to the vehicle group at both rostral and caudal points to SCI center (*p<0.05, n = 6/group).

We next examined the phenotype of BrdU labelled cells with multiple fluorescent labelling in the spinal cord sections of the rats treated with vehicle, ChABC, GFs and ChABC+GFs. To account for the possible overlapping of expression that exists among different cell types, we developed triple labelling paradigms to accurately identify the phenotype of BrdU labelled cells. Under basal injury conditions (i.e. vehicle-treated group), approximately 50% of BrdU labelled cells were GFAP+ (Fig. 5). Interestingly, we found a reduction in the number of newly generated astrocytes marked as GFAP+/BrdU+ under all treatment conditions compared to the vehicle-treated rats. This reduction was statistically significant for ChABC and ChABC+GFs in both rostral and caudal points to the injury center (Fig. 5A–E). Of note, our immunolabeling studies showed the absence of GFAP expression in the ependymal cells, which is in agreement with previous findings by Frisen group [7] that also showed the lack of GFAP expression in genetically labelled ependymal cells surrounding the central canal. Although GFAP positive astrocytes were closely surrounding the ependymal layer and sending their cell processes into the region, no GFAP expressing cell was observed inside the ependymal layer.

**Figure 6:**
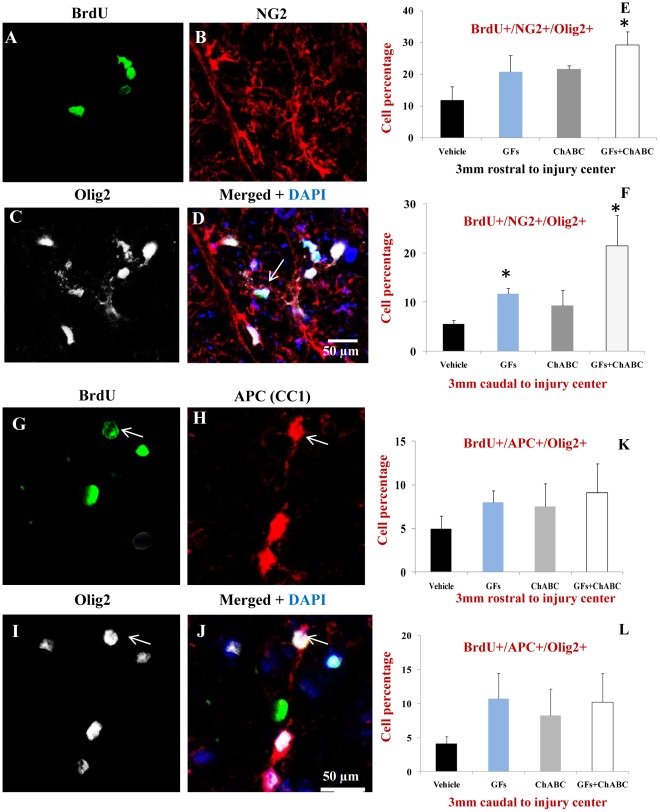
ChABC and GF treatments enhance the proliferation and differentiation of oligodendrocyte precursor cells (OPCs) after SCI. (A–D) Representative confocal images showing triple immunostaining for NG2, Olig2 and BrdU in injured spinal cord. To accurately identify OPCs among BrdU+ cells, we used double labelling for Olig2 and NG2 (Table 2). (E–F) Our quantification for BrdU+/Olig2+/NG2+ (arrows in D) revealed a significant increase in the number of OPCs after ChABC+GF treatments relative to vehicle (approximately a three-fold increase in 3 mm rostral point and a four-fold increase in 3 mm caudal point). (G–J) We also used BrdU/Olig2/APC immunostaining combination to mark and quantify mature oligodendrocytes after ChABC and GF treatments. Our analysis showed a positive trend for an increase in the number of newly generated mature oligodendrocytes under ChABC and/or GF treatments compared to vehicle at both 3 mm rostral and caudal points to the injury center (K–L). However, this increased level did not reach a statistically significant level. *p<0.05, n = 6/group.

**Figure 7:**
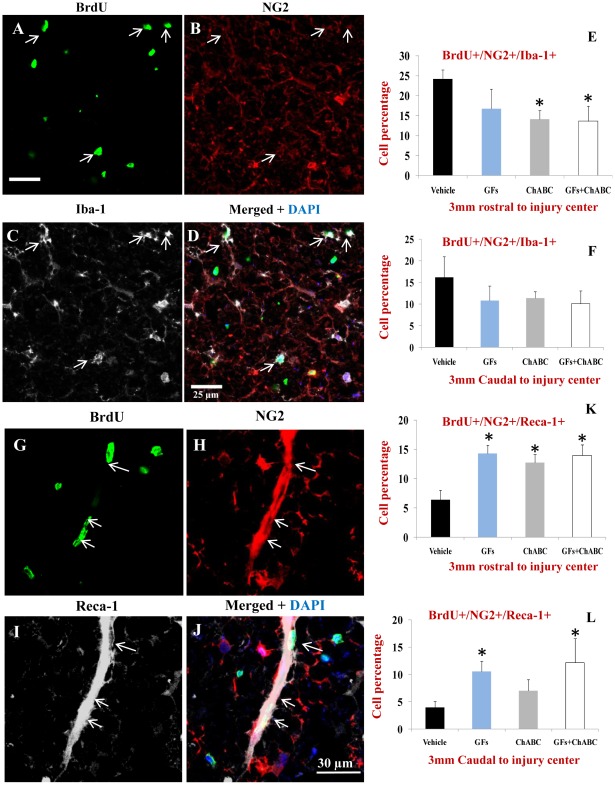
ChABC and GF treatments attenuate the proliferation of microglia/macrophages and promote the generation of new endothelial cells after SCI. (A–D) Representative confocal images of BrdU+/NG2+ macrophages/microglia marked with Iba-1 in the injured spinal cord (arrows). (E–F) Under baseline SCI condition, macrophages/microglia comprised about 25% and 17% of BrdU+/NG2+ cells in rostral and caudal points to the injury center, respectively. After treatment with ChABC and/or GFs, we found a reduction in the number of BrdU+/NG2+/IbA-1+ cells that was statistically significant for ChABC and ChABC+GFs treatment groups relative to the vehicle group. (G–J) Representative confocal images show newly generated endothelial cells marked by Reca-1 and NG2 among BrdU+ cells. Reca-1 positive endothelial cells comprised a subpopulation of proliferating NG2+ cells after SCI (J). (K–L) Quantification of BrdU+/NG2+/Reca-1+ cells showed a significant number of newly generated endothelial cells after treatment with ChABC and/or GFs at both rostral and caudal points to the injury center compared to the vehicle group. *p<0.05, n = 6/group.

The decrease in the number of new astrocytes was accompanied by an increase in the number of BrdU+ cells within the oligodendroglial lineage. Using triple fluorescent labelling for Olig2/GFAP/BrdU, we found a significant increase in the number of Olig2+/GFAP−/BrdU+ cells after treatment with GF and ChABC+GFs (Fig. 5F–K). Olig2 transcription factor has been shown to be also expressed by reactive astrocytes in some regions of the spinal cord after injury; therefore we used double labelling for Olig2 and GFAP to accurately identify newly generated oligodendrocytes marked as Olig2+/GFAP- among BrdU+ cells. We further determined the percentage of oligodendrocyte precursor cells (OPCs) and mature oligodendrocytes among the Olig2+/GFAP- population. We employed NG2/Olig2/BrdU and APC/Olig2/BrdU labelling combinations to distinguish OPCs from mature oligodendrocytes, respectively, as we previously described [18], [21], [28]. Our quantitative assessments revealed the presence of both immature and mature oligodendrocytes among the BrdU labelled cells with 2–3 folds more new OPCs (NG2+/Olig2+/BrdU+) than mature oligodendrocytes (APC+/Olig2+/BrdU+) oligodendrocytes (Fig. 6). Interestingly, the new NG2+/Olig2+ OPCs were only a small subpopulation of the total NG2+/BrdU+ cells after SCI. Under basal SCI condition, although we found that approximately 35–45% of the BrdU+ cells were expressing NG2 in 3 mm caudal and rostral points to the injury, only about 5–10% of the NG2+/BrdU+ cells were NG2+/Olig2+/BrdU+ OPCs. To determine the phenotype of the remaining NG2+ cells, we conducted triple immunostaining to include markers for macrophages/microglia as well as vascular endothelial cells. Our quantitative assessments indicated the presence of newly generated NG2+ endothelial cells (NG2+/Reca1+/BrdU) and macrophages/microglia (NG2+/Iba1+/BrdU+) under all experimental conditions suggesting that NG2+ cells represent multiple lineages in the spinal cord (Fig. 7). Interestingly, we found that ChABC and/or GF treatments significantly increased the number of newly generated endothelial cells after SCI (Fig. 7G–L) suggestive of a potential for these treatments in promoting angiogenesis. While both GFs and ChABC had competitive effects on endothelial cell proliferation, only ChABC and ChABC+GF treatments exerted a significant effect on attenuating the proliferation of macrophages/microglia in the penumbra regions of the SCI lesion (7A–F).

### Combination of ChABC and Growth Factors Attenuate Astrogliosis and Inflammation Around the SCI Lesion

**Figure 8:**
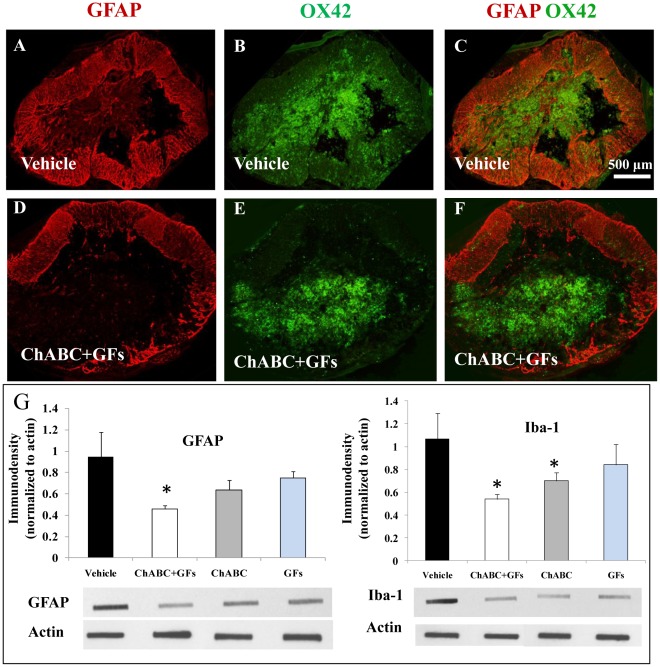
ChABC and GF treatments reduce the injury-induced astrogliosis and inflammation at the lesion site. (**A–F**) Cross sections of the spinal cord at the lesion site from vehicle and ChABC+GF treated animals show immunostaining for astrocytes (marked by GFAP) and macrophages/microglia (marked by OX42). Confocal images clearly show an overall reduction in the expression of GFAP and OX42 particularly in the surrounding paranchymal region in ChABC+GFs treated spinal cords relative to the vehicle group. Our immunoblotting analyses indicated a significant reduction in GFAP and Iba-1(macrophages/microglia) at the lesion site in ChABC+GF and ChABC and ChABC+GF treatments, respectively. *p<0.05, n = 4/group.

We further investigated the influence of ChABC and GF therapy on the evolution of reactive astrogliosis and activation of microglia/macrophages at the lesion site. Our quantitative slot blot analysis indicated a positive trend in reducing the expression of GFAP and Iba-1, marking astrocytes and macrophages/microglia, respectively, in all treated groups (Figure 8). However, only the group with ChABC+GF treatments showed a significant decrease in GFAP expression at the SCI center (Fig. 8). We also found a significant reduction in the presence of macrophages/microglia in the SCI lesion in groups treated with ChABC and ChABC+GFs (Fig. 8).

## Discussion

In the present study, to our knowledge, we demonstrate for the first time that inhibiting CSPGs combined with growth factor treatment enhances the regenerative response of endogenous spinal NPCs (esNPCs) in a clinically relevant model of compressive/contusive rat SCI. This strategy that included the administration of ChABC and a cocktail of growth factors bFGF, EGF and PDGF-AA increased the proliferation of esNPCs, reduced the number of new astrocytes and promoted the differentiation of esNPCs along an oligodendroglial lineage, a prerequisite for remyelination. Furthermore, the ChABC and GF therapy enhanced the response of non-neural cells by increasing the generation of new vascular endothelial cells and decreasing the number of proliferating macrophages/microglia after SCI. Collectively, our data provide strong evidence that the activity of esNPCs is hindered in their post-SCI niche and approaches to improve their environment would enhance the regenerative potential of esNPCs for the repair of SCI.

Following SCI, loss of oligodendrocytes [29] results in demyelination that not only leads to axonal dysfunction but also further impairs the integrity of surviving injured axons [1], [20], [25], [30], [31]. Emerging evidence from our group and others has established the promising potential of transplanting NPCs derived from the adult CNS tissues in oligodendrocyte replacement and remyelination associated with functional recovery after SCI [18], [21], [32], [33], [34]. Existence of resident NPCs in the ependyma of the spinal cord with the capacity to generate new glial cells [4], [6], [10], [35] has raised an exciting possibility for enhancing endogenous repair after SCI. Interestingly, recent evidence shows that the esNPCs indeed generate more oligodendrocytes than astrocytes under normal conditions in the spinal cord [6], [7]. However, despite this capacity, the majority of esNPCs differentiate into astrocytes after SCI and only a smaller number give rise to oligodendrocytes [6]. In a dorsal funiculi SCI by using genetic labelling of esNPCs in the ependyma, Frisen group has shown that the newly generated astrocytes become activated and recruited to the site of SCI and contribute to astrogliosis and glial scar [6]. This evidence suggests that the response of esNPCs to SCI is not supportive; and on the contrary is inhibitory to the regenerative activities inside the injured spinal cord. For example, the inhibitory influence of astrogliosis on limiting axonal regeneration and plasticity after SCI is very well established [3], [16], [17], [36], [37], [38], [39], [40]. Given this, it is therefore critical to optimize the behaviour of esNPCs in their post-SCI environment not only to promote endogenous repair process, but also to reverse the otherwise non-constructive response of esNPCs after SCI.

Previous studies have shown that *in vivo* administration of EGF and bFGF, two growth factors essential for the expansion and maintenance of NPCs *in vitro*; increases the proliferation of ependymal cells after SCI [41], [42]. However, despite their increased activity, the generation of new oligodendrocytes from esNPCs was unaffected after SCI [41], [42], suggesting that further optimization is needed to support oligodendrocyte differentiation. Another study showed that combined overexpression of Neurogenin2 and Mash1 enhanced the generation of new neurons and oligodendrocytes at the expense of astrocytes after SCI [43]; however this strategy did not afford to support the maintenance of the newly generated oligodendrocytes. This observation implicates the inhibitory influence of SCI microenvironment on the integration and survival of newly differentiated cells within the injured spinal cord. Our group has recently demonstrated a key role for the SCI-induced up-regulation of CSPGs in the glial scar on the survival and integration of transplanted NPCs *in vivo* [18]. We showed that degradation of CSPGs with ChABC significantly improved the migration of transplanted NPCs from the implantation sites to the SCI lesion. ChABC treatment also enhanced the long-term survival of transplanted NPCs and aided in their integration with the spinal cord axons. We combined ChABC with administration of EGF, bFGF and PDGF-AA to enhance the survival of transplanted NPCs and direct their fate into oligodendrocytes. PDGF-AA is growth factor secreted by astrocytes in the CNS that promotes the proliferation of neural progenitors and stimulates the differentiation of oligodendrocytes [44], [45], and is also implicated in the survival of and migration of newly formed oligodendrocytes [46], [47]. Moreover, PDGF, in synergy with bFGF, regulates the proliferative response of adult oligodendrocyte progenitors [48], [49], [50]. In our transplantation studeis, combination of these growth factors afforded to direct the majority of transplanted NPCs (about 80%) to differentiate along an oligodendroglial lineage [18]. Interestingly, although ChABC alone did not show any additional benefits to the effects of growth factors in enhancing the differentiation of oligodendrocytes, it markedly increased the migration, survival and integration of transplanted NPCs compared to the groups that only received growth factors. Our data, therefore; indicate the importance of combinatorial approaches aimed at enhancing the proliferation and differentiation of NPCs as well as optimizing their integration within the injured spinal cord.

In the present study, we examined the combined effects ChABC and GFs on the cellular response of esNPCs after SCI. We administered ChABC at day 4 following SCI, when dense deposits of CSPGs have begun to accumulate around the SCI lesion. We also combined ChABC treatment with a cocktail of EGF, bFGF and PDGF-AA. We undertook continuous BrdU incorporation during the treatment period to label proliferating cells. Here, we focused on characterizing the response of esNPCs to ChABC and GFs at the end of treatments (i.e. eight days after the treatments or 12 days after SCI). Overall, we found a significant increase in the number of proliferating cells in the ChABC treated group compared to the vehicle-treated counterparts. The effect of ChABC alone was comparable to the effects of GF treatment alone. Importantly, the increased number of BrdU positive cells reflected an increase in the number of activated esNPCs (Nestin+/GFAP−/BrdU+) after GF and/or ChABC treatments. Interestingly, ChABC and GF treatments reduced the number of new astrocytes and increased the generation of new oligodendrocytes after SCI. Although ChABC and GFs exerted similar effects on attenuating astrocytic differentiation, the presence of GFs in this therapeutic combination was indispensable in directing the fate of esNPCs toward an oligodendroglial lineage (BrdU+/Olig2+/GFAP-). This observation is also consistent with our previous studies in NPC transplantation approaches that showed no significant effect of ChABC on oligodendrocyte differentiation of transplanted NPCs after SCI [18], [21]. This evidence suggests that although ChABC does not appear to directly affect oligodendrocyte differentiation of esNPCs, it reduces their differentiation into astrocytes. While the exact molecular mechanisms by which CSPGs influence the fate of esNPCs after SCI require further elucidation, it seems that up-regulation of CSPGs by activated astrocytes create a negative feedback that results in exacerbation of astrogliosis.

In our studies, in agreement with previous reports [5], [51], [52], we found a significant number of newly generated NG2 expressing cells. NG2 cells represent a heterogeneous population after SCI that include oligodendrocyte precursor cells (OPCs), macrophages/microglia and endothelial cells [9], [53], [54], [55]. Although the overall number of NG2 expressing cells remained unchanged in ChABC and/or GF treated groups compared to the vehicle treated group, we found a significant difference in the phenotype of NG2 cells under different treatments. Animals treated with ChABC+GFs, showed a significantly increased number of NG2+/Olig2+/BrdU+ cells which suggest the synergistic effects of ChABC and GFs in promoting differentiation of esNPCs into OPCs after SCI. We also observed a significant increase in the number of newly generated endothelial cells under both GFs and ChABC treatments compared to the baseline injury with no further benefits of their combination. On the contrary, the number of NG2 expressing macrophages/microglia was significantly reduced only in the groups treated with ChABC and ChABC+GFs with no additional effects from GF therapy. Our assessment of SCI epicentre also revealed a reduction in the presence of macrophages/microglial after ChABC and ChABC+GFs treatments. This evidence suggests a role for ChABC treatment in attenuating the inflammatory response after SCI. Further elucidation is required to understand the mechanisms by which ChABC affects the activation of inflammatory cells. We also found a positive trend in increased number of new mature oligodendrocytes (APC+/Olig2+/BrdU+) under GFs and/or ChABC treatments; however, it did not reach a significant level at 12 days post-SCI. It is possible that either these treatments are not sufficient to fully support oligodendroglial maturation in the spinal cord or more time is needed for transitioning from an OPC phenotype to a mature myelinating oligodendrocyte phenotype. Long-term investigations are required to determine whether these treatments can promote oligodendrocytes maturation after SCI. As expected, no neuronal differentiation was observed among BrdU labelled cells which is in complete agreement with our previous studies and those of others that show NPCs in the environment of adult spinal cord are mostly capable of generating glial cells [6], [7], [18], [21]. Apparently, administration of ChABC and GFs did not afford to stimulate neurogenesis.

### Conclusions

In the present work, we have identified another mechanism by which CSPGs hinder the repair of SCI. For a number of years, CSPGs were mainly considered inhibitory for axonal regeneration. Here, we report for the first time that perturbing the inhibitory properties of CSPGs in the post-SCI environment combined with the administration of growth factors EGF, bFGF and PDGF-AA significantly enhanced the response of endogenous spinal cord NPCs. This strategy effectively increased the proliferation of esNPCs and enhanced their differentiation into OPCs similar to their differentiation pattern seen under normal condition. Our results also show that perturbing CSPGs and increasing the level of GFs has the potential to positively improve the microenvironment of SCI by reducing inflammation and facilitating the reconstruction of compromised vasculature after SCI. Our data provide support for the promising effects of this combinatorial approach, which addresses several key pathomechanisms, for the repair of SCI.

## Supporting Information

Table S1 **Summary of** ***in vivo*** **experiments and experimental groups.**
(DOCX)
