# Supplementary material for: Chondroitinase and Growth Factors Enhance Activation and Oligodendrocyte Differentiation of Endogenous Neural Precursor Cells after Spinal Cord Injury
Source: PLoS One. 2012 May 22;7(5):e37589. doi: 10.1371/journal.pone.0037589 (PMC3358255; doi:10.1371/journal.pone.0037589)
Supplement: Table S1 — Summary of in vivo experiments and experimental groups. (DOCX) [file pone.0037589.s001.docx]

| **Table S1. Summary of *in vivo* experiments and experimental groups** | | | | | | | | | | | | |
| --- | --- | --- | --- | --- | --- | --- | --- | --- | --- | --- | --- | --- |
| **SCI Treatment begins Treatment ends** | | | | | | | | | | | | |
| **0** | 1 | 2 | 3 | **4** | 5 | 6 | 7 | 8 | 9 | 10 | **11** | 12 |
| **Days after SCI ------------------------------------------------------**  **Days after SCI** | | | | | | | | | | | | |
| **Daily BrdU injection** | | | | | | | | | | | | |
| **Treatment Groups:**   - **Vehicle:** Bovine serum albumin (BSA) in saline - **Growth factors (GFs)** - **Chondroitinase ABC** (ChABC) - **ChABC + GFs** | | | | | | | | | | | | |
